# Benchmark Study on the Smallest Bimolecular Nucleophilic Substitution Reaction: H^−^+CH_4_ → CH_4_+H^−^

**DOI:** 10.3390/molecules18077726

**Published:** 2013-07-03

**Authors:** Marcel Swart, F. Matthias Bickelhaupt

**Affiliations:** 1 Institució Catalana de Recerca i Estudis Avançats (ICREA), Pg. Lluís Companys 23, 08010 Barcelona, Spain; 2 Institut de Química Computacional i Catàlisi (IQCC) and Departament de Química, Universitat de Girona, Campus Montilivi, 17071 Girona, Spain; 3 Department of Theoretical Chemistry & Amsterdam Center for Multiscale Modeling, VU University, De Boelelaan 1083, 1081 HV Amsterdam, The Netherlands; E-Mail: f.m.bickelhaupt@vu.nl; 4 Institute of Molecules and Materials, Radboud University Nijmegen, Heyendaalseweg 135, 6525 AJ Nijmegen, The Netherlands

**Keywords:** S_N_2 reaction, density functional theory, benchmark study, coupled cluster theory, bimolecular substitution, gas phase reactivity

## Abstract

We report here a benchmark study on the bimolecular nucleophilic substitution (S_N_2) reaction between hydride and methane, for which we have obtained reference energies at the coupled cluster toward full configuration-interaction limit (CC-cf/CBS). Several wavefunction (HF, MP2, coupled cluster) and density functional methods are compared for their reliability regarding these reference data.

## 1. Introduction

**Figure 1:**
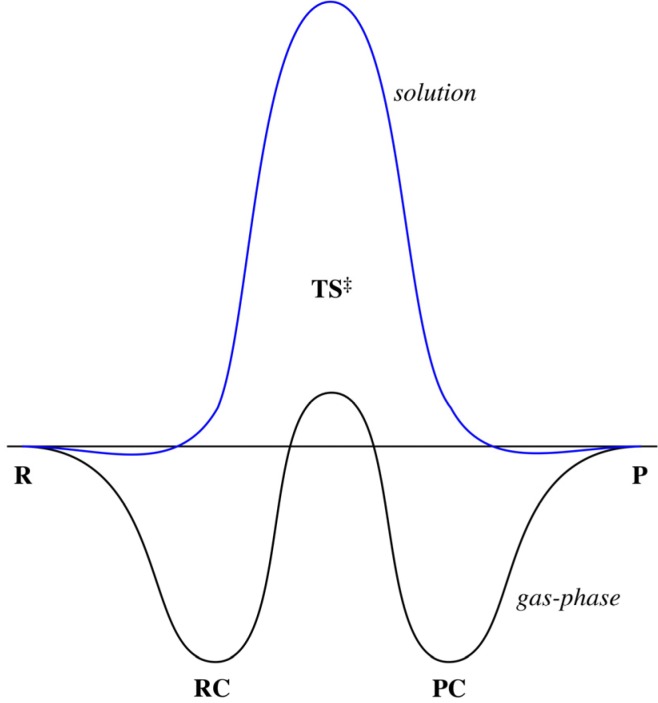
Energy profile for S_N_2 reaction in gas phase and in solution.

Bimolecular substitution (S_N_2) reactions play an important role in organic chemistry and in biochemistry (DNA replication mechanism). Interestingly, there is a profound solvent effect present which has a major effect on the reaction barriers and intermediates. For example, the prototypical S_N_2 reaction of chloride with methyl chloride shows in the gas phase a double-well potential (see Figure 1) with deep wells and a reduced barrier. On the other hand, in solution the energy profile turns basically into a unimodal reaction [1,2,3,4,5,6,7,8,9,10,11,12] (see Figure 1), accompanied by a significant increase of the reaction barrier. In previous studies [13,14,15,16,17,18,19,20,21,22,23,24,25,26,27,28,29,30,31,32,33,34,35,36,37,38,39,40,41,42] it was shown that coupled cluster methods in general give accurate results for the energy profile of S_N_2 reactions, while density functional methods give qualitatively correct results but often underestimate barriers [15]. This has led to the design of new and improved density functionals (SSB-D [43], S12g [44] and S12h [44]), where in particular the latter hybrid functional (S12h) was shown to provide accurate results for the complete energy profile of S_N_2 reactions.

Here we have studied the smallest S_N_2 reaction possible, between hydride and methane:


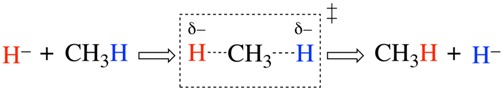


For this reaction, we have been able to perform coupled cluster calculations [45] up to the level of CCSDT/aug-cc-pVTZ, and through extrapolation techniques we have obtained reference data at CC-cf/CBS (CC-cf=continued fraction [46] extrapolation toward full-CI limit; CBS = Complete Basis Set). Moreover, we have explored the energy profile for this reaction with 28 density functionals, (LDA, [47,48,49] PBE, [50] PBE-D_3_, [50,51] PBE0, [52,53,54] PBE0-D_3_, [51,52,53,54] PW91, [55,56] BP86, [57,58] revPBE, [59] OPBE, [50,60,61] OLYP, [61,62] B3LYP, [63,64] B3LYP-D_3_, [51,63,64] BLYP, [57,62] B2PLYP, [65] M06, [66] M06-2X, [66] M06-L, [67] B97, [68] B97-3, [69] B97-D_2_, [51] TPSS, [70] TPSS-D_3_, [51,70] TPSSh, [71] SSB-D, [43] S12g, [44] S12h, [44] CAM-S12h, [44] CAM-B3LYP [72]), among which the most popular ones from the DFT2012 popularity poll [73] and the newly developed S12g/S12h functional [44].

## 2. Results and Discussion

The complete energy profile for the S_N_2 reaction of H^−^+CH_4_ was studied using both wavefunction and density functional methods. The reaction proceeds from the reactants (R, see Figure 1) towards a reactant complex (RC) and then crosses the central barrier (TS) to reach a product complex (PC) and finally products (P). The RC is reached early on, e.g., with a C-H(nucleophile) distance of some 3.84 Å; this is ca. 0.7 Å longer than the case of Cl^−^ + CH_3_Cl (3.15 Å) even though the size of the nucleophile is probably much smaller here [*note however that Pauling* [74] *reported a larger ionic radius for H^−^ (2.08 Å) than for Cl^−^ (1.81 Å), while Frecer* [75,76] *through Monte Carlo obtained values of 2.28 Å (H^−^) and 2.30 Å (Cl^−^) respectively*]. This is consistent with a very weak ion-molecule interaction in the reactant complexes. Interestingly enough, at the highest level for which we could obtain the energies directly, CCSDT/atz (see Table 1), this leads to an energy profile of this gas-phase reaction that is more reminiscent of an S_N_2 profile in solution [1,2,77] (see Figure 1). For example, the RC well is almost non-existent with a depth of 0.9 kcal·mol^−1^ and the barrier is quite steep with a value of 49.4 kcal·mol^−1^.

### 2.1. Coupled Cluster Results

We extrapolated the coupled cluster energies to come as close as possible to the full-CI result, for which we use the continued-fraction approximant [46]. There are two possibilities for this extrapolation (Equations 1a,b), using either the CCSD(T) energy (as we do here in this first part), or the CCSDT energy (as discussed later on), for the *δ*_3_ ingredient. The resulting *E_CC-cf_* energies are also given in Table 1:


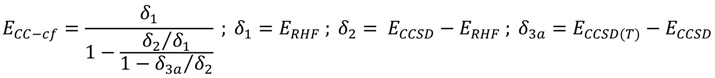
(1a)


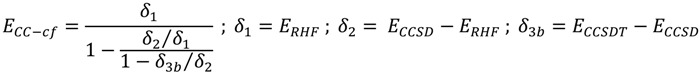
(1b)

The results of Table 1 make it clear that there is a clear basis set effect, where it is not really important to increase the basis set size but it is more important to include diffuse functions [42,78]. Given that we deal here with anionic species, for which diffuse functions are important [21,42,79], this comes as no surprise. There is also a significant electron-correlation effect, where energies obtained at the CCSDT level do not seem to have converged to the full-CI limit. For instance, the well depth increases with the atz basis from −0.27 kcal·mol^−1^ (RHF) to −0.69 kcal·mol^−1^ at CCSD, −0.90 kcal·mol^−1^ at CCSD(T), −0.93 kcal·mol^−1^ at CCSDT and −1.22 kcal·mol^−1^ at CC-cf. Likewise, the barrier continues to drop from 62.6 kcal·mol^−1^ at RHF to 49.4 kcal·mol^−1^ at CCSDT, and reaches 49.0 kcal·mol^−1^ at CC-cf. It should be noted that the perturbative triples approach in CCSD(T) gives a good approximation for the CCSDT energies (difference 0.05−0.30 kcal·mol^−1^). Based on the extrapolation towards full-CI and complete basis set with CC-cf at increasingly larger basis sets (CC-cf/CBS), final reference energies of -1.20 kcal·mol^−1^ (RC) and +48.90 kcal·mol^−1^ (TS) are obtained.

**Table 1:** Relative energies (kcal·mol^−1^) obtained with wavefunction and density functional methods ^a^.

	dz	tz	qz	adz	atz	aqz	dz	tz	qz	adz	atz	aqz
	RC	RC	RC	RC	RC	RC	TS	TS	TS	TS	TS	TS
RHF	−2.55	−2.02	−1.70	−0.27	−0.27	−0.27	52.90	56.86	58.47	61.98	62.59	62.61
MP2	−3.41	−3.15	−2.93	−0.87	−0.91	−0.87	43.89	46.44	47.28	49.36	50.19	50.21
CCSD	−3.53	−3.26	−2.99	−0.70	−0.69	−0.62	42.23	45.95	47.61	51.87	52.97	53.03
CCSD(T)	−3.66	−3.46	−3.23	−0.88	−0.90	−0.85	40.20	43.41	44.73	48.88	49.67	49.63
CCSDT	−3.68	−3.49	n/a^b^	−0.92	−0.93	n/a ^b^	39.92	43.10	44.41	48.61	49.42	n/a^b^
CC−cf ^c^	−3.80	−3.71	−3.53	−1.13	−1.22	−1.19	39.83	42.85	44.07	48.30	48.95	48.87
LDA	−10.07	−7.78	−6.86	−3.08	−2.77	−2.74	22.86	28.01	29.83	33.26	34.12	34.16
PBE	−7.72	−5.69	−5.14	−1.99	−1.85	−1.83	27.68	32.76	34.49	38.26	39.00	39.09
PBE−D_3_	−8.01	−5.98	−5.40	−2.21	−2.05	−2.03	27.43	32.51	34.25	38.05	38.80	38.88
PBE0	−5.56	−4.18	−3.69	−1.18	−1.08	−1.07	35.04	39.61	41.21	44.38	45.03	45.08
PBE0−D_3_	−5.84	−4.43	−3.92	−1.36	−1.25	−1.24	34.77	39.35	40.96	44.16	44.81	44.86
PW91	−7.97	5.95	−5.49	−2.46	−2.37	−2.37	27.32	32.46	34.12	37.76	38.52	38.59
BP86	−6.50	−4.39	−3.74	−0.78	−0.65	−0.62	28.34	33.58	35.49	39.31	40.24	40.32
revPBE	−6.48	−4.64	−4.23	−1.54	−1.50	−1.51	30.16	35.20	36.87	40.62	41.29	41.38
OPBE	−4.72	−3.53	−3.34	n/a ^d^	n/a ^d^	n/a ^d^	34.49	38.87	40.18	43.01	43.22	43.28
OLYP	−6.28	−4.77	−4.57	n/a ^d^	n/a ^d^	n/a ^d^	33.46	38.29	39.71	43.28	43.82	43.92
B3LYP	−5.73	−4.02	−3.50	−0.78	−0.73	−0.72	34.40	39.49	41.20	44.78	45.84	45.91
B3LYP−D_3_	−6.12	−4.35	−3.80	−0.98	−0.91	−0.89	33.91	39.03	40.75	44.39	45.46	45.53
BLYP	−7.34	−5.02	−4.44	−1.22	−1.18	−1.18	28.63	34.32	36.17	40.35	41.56	41.66
B2PLYP	−4.21	−3.00	−2.51	+0.02	+0.03	+0.04	38.49	42.52	43.97	47.40	48.46	48.53
M06	−5.54	−3.99	−3.92	−1.80	−1.84	−2.01	36.87	41.94	42.50	46.29	46.56	46.13
M06−2X	−5.61	−4.43	−2.42	−1.26	−1.10	−1.06	36.08	41.35	43.58	45.16	47.11	46.95
M06−L	−5.46	−4.27	−3.76	−1.27	−1.19	−1.18	39.80	44.00	45.67	48.79	49.35	49.45
B97	−5.72	−4.30	−3.84	−1.24	−1.15	−1.11	34.31	38.99	40.70	44.14	44.96	45.11
B97−3	−4.78	−3.55	−3.00	−0.63	−0.51	−0.44	37.68	41.99	43.82	47.32	48.26	48.46
B97−D_2_	−6.57	−4.62	−4.14	−1.33	−1.21	−1.20	28.69	33.93	35.74	40.05	41.03	41.08
TPSS	−5.58	−4.10	−3.69	−1.38	−1.33	−1.32	30.75	35.82	37.35	40.33	41.27	41.30
TPSS−D_3_	−5.98	−4.44	−4.00	−1.60	−1.51	−1.50	30.37	35.47	37.00	40.01	40.97	40.99
TPSSh	−5.05	−3.73	−3.33	−1.12	−1.07	−1.06	33.34	38.25	39.76	42.61	43.51	43.52
SSB−D	−7.89	−6.12	−5.54	−2.15	−2.04	−2.01	30.65	35.04	36.82	40.55	41.08	41.20
S12g	−8.20	−6.36	−5.77	−2.33	−2.18	−2.15	30.36	34.95	36.74	40.51	41.10	41.21
S12h	−6.18	−4.74	−4.18	−1.47	−1.37	−1.35	36.51	40.99	42.66	45.97	46.60	46.67
CAM−S12h	−5.71	−4.38	−3.83	−1.31	−1.22	−1.21	38.35	42.83	44.50	47.72	48.35	48.41
CAM−B3LYP	−5.03	−3.62	−3.09	−0.64	−0.61	−0.60	38.85	43.88	45.61	49.11	50.03	50.08

^a^ energies relative to reactants, for each method at their own optimized geometry; ^b^ not available due to insufficient computational resources; ^c^ obtained with equation 1a at CCSD(T) optimized geometry; ^d^ not available due to dissociation towards reactants, *i.e.*, no RC−complex found.

The results obtained with the different levels of coupled cluster method, *i.e.*, CCSD, CCSD(T), CCSDT and CC-cf, together with RHF and MP2 (see Table 1) indicate that CCSD may be sufficient for getting good results. CCSD underestimates the RC well depth (e.g., by 0.3−0.6 kcal·mol^−1^), and overestimates the barrier by ca. 3−4 kcal·mol^−1^. MP2 works in this respect even better with deviations from CC-cf that are twice as small, even though the computational effort is more or less the same as CCSD. As already noted often before, RHF cannot be trusted for these energy profiles as it gives barriers which are too large.

### 2.2. Density Functional Energies

All density functionals show the correct energy profile with a shallow well for the RC (−0.4 to −2.7 kcal·mol^−1^ with the largest basis set aqz), and a substantial barrier (34.2 to 50.1 kcal·mol^−1^ with the aqz basis). Nevertheless, the results for the 28 density functionals show quite a diversity in the accuracy for the energy profile, even though some general trends are obvious: they tend to overestimate the RC well depth, and (generally) underestimate the reaction barrier. The least reliable functional is not surprisingly LDA, which for the RC predicts a well depth of −2.74 kcal·mol^−1^, and places the TS at +34.16 kcal·mol^−1^ (e.g., a deviation of ca. 15 kcal·mol^−1^). Early GGA functionals (PBE, BP86 [80], BLYP) improve the barrier by ca. 5–7 kcal·mol^−1^, and a further 3 kcal·mol^−1^ is obtained by the use of OPTX in OPBE/OLYP. Surprisingly, SSB−D predicts a barrier that is ca. 2.0 kcal·mol^−1^ lower than OPBE, even though prior studies showed them to behave similarly. This cannot be due to the inclusion of dispersion in SSB−D, since the effect of including Grimme’s dispersion energy is limited (< 0.5 kcal·mol^−1^, see Table 1). Even better results are obtained with hybrid functionals and reasonable results are obtained: the difference with the CC-cf results is now only 3−4 kcal·mol^−1^ (*at a fraction of the computational cost*) for the most often used hybrid functionals (B3LYP, PBE0, M06). The recently developed S12h and M06−2X bring the deviation from CC−cf down to ca. 2 kcal·mol^−1^, while excellent results (deviation <0.5 kcal·mol^−1^) are obtained with four functionals: B2PLYP (48.53 kcal·mol^−1^), M06−L (49.45 kcal·mol^−1^), B97−3 (48.46 kcal·mol^−1^) and CAM−S12h (48.41 kcal·mol^−1^). Of these four, two give an excellent description of the RC well depth: M06−L (−1.18 kcal·mol^−1^) and CAM−S12h (−1.21 kcal·mol^−1^), while the other two underestimate it slightly (B97−3, −0.44 kcal·mol^−1^) or show a non-existent RC (B2PLYP, +0.04 kcal·mol^−1^). The non−existence of a RC happens also for OPBE and OLYP with the augmented basis set (adz, atz, aqz), where the optimization proceeds towards reactants.

### 2.3. Structural Parameters

All methods used here confirm the early character of the RC, with a distance between the nucleophile (Nu) and the central carbon observed within the range of 2.97 Å (B2PLYP) to 4.73 Å (RHF) (see Table 2). These two values are, together with LDA (3.06 Å), rather different from the other wavefunction and density functional methods that give values roughly between 3.4 and 4.0 Å. Moreover, this C−Nu distance is the one that distinguishes the several methods for the deviations with respect to the CCSDT/atz results. The variation for the other structural parameters is much smaller (see Table 2).

**Table 2:** Structural parameters (Å, °) for stationary points, obtained with atz basis set.

	r(C−H) ^a^	r(C−LG) ^b^	r(C−Nu) ^c^	r(C−H) ^d^	℘(H−C−LG) ^e^	r(C−LG) ^f^	r(C−H) ^g^	*MAD ^h^*
RHF	1.082	1.085	4.734	1.081	110.05	1.690	1.059	*0.898*
MP2	1.084	1.088	3.790	1.084	110.64	1.578	1.067	*0.073*
CCSD	1.086	1.090	4.039	1.086	110.41	1.629	1.067	*0.201*
CCSD(T)	1.088	1.092	3.857	1.087	110.55	1.629	1.069	*0.020*
CCSDT	1.088	1.092	3.838	1.087	110.56	1.633	1.069	*0*
LDA	1.097	1.104	3.055	1.098	111.60	1.570	1.081	*0.786*
PBE	1.096	1.101	3.525	1.096	110.79	1.609	1.078	*0.314*
PBE−D_3_	1.096	1.102	3.394	1.096	110.91	1.612	1.079	*0.444*
PBE0	1.089	1.093	3.586	1.089	110.74	1.603	1.071	*0.253*
PBE0−D_3_	1.089	1.093	3.473	1.089	110.83	1.606	1.072	*0.366*
PW91	1.094	1.099	3.523	1.094	110.76	1.614	1.076	*0.315*
BP86	1.096	1.100	3.862	1.096	110.54	1.620	1.079	*0.033*
revPBE	1.097	1.101	4.085	1.097	110.36	1.624	1.079	*0.248*
OPBE	1.094	n/a^i^	n/a^i^	n/a^i^	n/a^i^	1.571	1.078	*n/a^i^*
OLYP	1.093	n/a^i^	n/a^i^	n/a^i^	n/a^i^	1.604	1.075	*n/a^i^*
B3LYP	1.088	1.092	3.792	1.088	110.52	1.637	1.069	*0.046*
B3LYP−D_3_	1.089	1.093	3.565	1.088	110.67	1.642	1.069	*0.272*
BLYP	1.094	1.099	4.100	1.094	110.32	1.652	1.075	*0.263*
B2PLYP	1.093	1.100	2.967	1.093	111.50	1.559	1.079	*0.874*
M06	1.087	1.091	3.643	1.087	110.57	1.621	1.071	*0.195*
M06−2X	1.087	1.091	3.309	1.087	110.93	1.612	1.069	*0.529*
M06−L	1.085	1.088	4.088	1.085	110.33	1.646	1.069	*0.251*
B97	1.091	1.095	3.684	1.090	110.60	1.622	1.072	*0.154*
B97−3	1.087	1.091	3.789	1.087	110.50	1.612	1.069	*0.053*
B97−D_2_	1.095	1.100	3.892	1.095	110.46	1.672	1.077	*0.069*
TPSS	1.092	1.097	3.709	1.092	110.49	1.660	1.075	*0.238*
TPSS−D_3_	1.092	1.098	3.490	1.092	110.64	1.645	1.073	*0.348*
TPSSh	1.090	1.094	3.713	1.089	110.51	1.636	1.071	*0.125*
SSB−D	1.087	1.093	3.459	1.087	110.79	1.567	1.072	*0.384*
S12g	1.093	1.098	3.384	1.093	110.92	1.580	1.076	*0.457*
S12h	1.087	1.092	3.400	1.087	110.89	1.600	1.070	*0.439*
CAM−S12h	1.087	1.091	3.417	1.086	110.87	1.606	1.069	*0.421*
CAM−B3LYP	1.087	1.090	3.724	1.086	110.61	1.635	1.067	*0.114*

^a^ C−H distance in methane−reactant; ^b^ C−LG distance in RC, LG=leaving group; ^c^ C−Nu distance in RC, Nu=nucleophile; ^d^ C−H distance in RC; ^e^ angle H−C−LG in RC; ^f^ C−LG (C−Nu) distance at TS; ^g^ C−H distance at TS; ^h^ mean absolute deviation of distances compared to CCSDT/atz values; ^i^ not available due to dissociation towards reactants, *i.e.*, no RC−complex found.

### 2.4. Single−Point Calculations at CCSDT/atz Geometry

The comparison of the energy profiles for the different methods is of course influenced to some extent by the different geometries used with the different methods. Therefore, and in order to make an honest comparison between the different methods we also performed single-point energy calculations using the CCSDT/atz geometries. Given in Table 3 are the results for all wavefunction and density functional methods.

**Table 3:** Energy profile (aqz basis, kcal·mol^−1^) using single−point calculations at CCSDT/atz geometry.

Method	RC	TS		Method	RC	TS
RHF	0.04	63.01		B3LYP−D_3_	−0.89	45.54
MP2	−0.86	50.52		BLYP	−1.09	41.66
CCSD	−0.60	53.03		B2PLYP	−0.68	47.82
CCSD(T)	−0.85	49.63		M06	−2.01	46.15
CC-cf	−1.19	48.87		M06−2X	−1.02	47.01
				M06−L	−1.01	48.70
LDA	−2.35	34.62		B97	−1.11	45.14
PBE	−1.81	39.17		B97−3	−0.44	48.51
PBE-D_3_	−1.97	38.96		B97−D_2_	−1.16	41.18
PBE0	−1.07	45.18		TPSS	−1.31	41.29
PBE0-D_3_	−1.22	44.95		TPSS−D_3_	−1.49	40.99
PW91	−2.35	38.64		TPSSh	−1.05	43.52
BP86	−0.58	40.35		SSB−D	−1.99	41.65
revPBE	−1.39	41.39		S12g	−2.10	41.54
OPBE	−1.04	43.73		S12h	−1.32	46.79
OLYP	−1.79	44.03		CAM−S12h	−1.17	48.50
B3LYP	−0.69	45.92		CAM−B3LYP	−0.59	50.09

By comparing the results from Table 3 with those from Table 1, it can be seen that the influence of the geometry is limited. The largest difference for the different methods observed is of the order of 0.4 kcal·mol^−1^ (e.g., for LDA, −2.74 kcal·mol^−1^ for the RC at the LDA geometry, and −2.35 kcal·mol^−1^ at the CCSDT/atz geometry). However, the typical deviation is less than 0.1 kcal·mol^−1^ (*i.e.*, chemical accuracy); for instance, PBE−D_3_ shows differences of 0.06 and 0.08 kcal·mol^−1^ for the RC and TS energies with the two different geometries. All of this indicates that the energy surface is quite flat, as was already obvious from Figure 1.

### 2.5. Competition with Proton Transfer Pathway

An alternative reaction is possible in which the hydride abstracts a proton from methane. This leads to the formation of a methyl anion and H_2_:


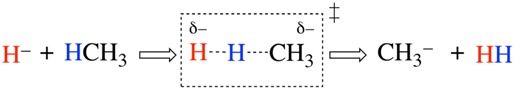


This alternative process is however associated with an endothermic reaction energy of +21.35 kcal·mol^−1^ at S12h/aqz. This pathway is beyond the scope of the present investigation which focuses on the thermoneutral identity S_N_2 reaction.

## 3. Experimental

All wavefunction based calculations (Hartree-Fock, Second-order Møller-Plesset Perturbation Theory, Coupled Cluster Theory) have been performed with the Coupled-Cluster techniques for Computational Chemistry (CFOUR) [81,82] program version 1.2, using a variety of Dunning’s correlation-consistent basis sets [83,84]: cc−pVXZ (X=D, T, Q, *abbreviated as dz, tz and qz respectively*) and aug−cc−pVXZ (X=D, T, Q, *abbreviated as adz, atz and aqz respectively*). The continued-fraction approximant [46] for obtaining coupled−cluster energies toward the full configuration-interaction limit (CC−cf) has been used with equations 1a,b to reach completeness of electron-correlation energies. The NWChem program [85] version 6.1 was used for all density functional calculations.

## 4. Conclusions

We have performed a benchmark study on the smallest bimolecular nucleophilic substitution (S_N_2) reaction possible: H^−^+CH_4_→ CH_4_+H^−^, for which we obtained reference data at the near-full-CI coupled cluster limit using the continued−fraction approximant (CC-cf). Unlike typical S_N_2 reactions in the gas phase, which usually show a double-well potential, the current reaction shows an energy profile that resembles more the unimodal profile of the S_N_2 reaction in solution, with a relatively shallow reactant-complex well of only −1.19 kcal·mol^−1^ and a high barrier amounting to 48.87 kcal·mol^−1^. All other computational methods also clearly show the steep reaction barrier that needs to be overcome (34−50 kcal·mol^−1^), and the very shallow wells of the (ion-dipole) reactant complex. All density functionals have the tendency to underestimate the reaction barrier, while for the RC the deviations compared to CC-cf are much smaller (< 0.5 kcal·mol^−1^). Excellent results have been obtained with B97-3, M06-L and the newly developed CAM−S12h functional.
